# Minimizing Glucose Excursions (GEM) With Continuous Glucose Monitoring in Type 2 Diabetes: A Randomized Clinical Trial

**DOI:** 10.1210/jendso/bvaa118

**Published:** 2020-08-18

**Authors:** Daniel J Cox, Tom Banton, Matthew Moncrief, Mark Conaway, Anne Diamond, Anthony L McCall

**Affiliations:** 1 University of Virginia School of Medicine, Center for Behavioral Medicine Research, Charlottesville, Virginia; 2 University of Virginia School of Medicine, Public Health Sciences, Charlottesville, VA; 3 University of Virginia School of Medicine, Endocrinology and Metabolism, Charlottesville, VA

**Keywords:** type 2 diabetes mellitus, glycemic load, exercise, physical activities, continuous glucose monitoring, postprandial blood glucose

## Abstract

This study aimed to compare conventional medication management of type 2 diabetes (T2D) to medication management in conjunction with a lifestyle intervention using continuous glucose monitoring to minimize glucose excursions. Thirty adults (63% female; mean age, 53.3 years) who were diagnosed with T2D for less than 11 years (mean, 5.6 years), had glycated A_1c_ (HbA_1c_) ≥ 7.0% (51 mmol/mol) (mean 8.8%, [73 mmol/mol]), and were not using insulin, were randomly assigned in a 1:2 ratio to routine care (RC) or 4 group sessions of glycemic excursion minimization plus real-time CGM (GEM^CGM^). Assessments at baseline and 5 months included a physical exam, metabolic and lipid panels, a review of diabetes medications, and psychological questionnaires. For the week following assessments, participants wore a blinded activity monitor and completed 3 days of 24-hour dietary recall. A subgroup also wore a blinded CGM. GEM^CGM^ participants significantly improved HbA_1c_ (from 8.9% to 7.6% [74-60 mmol/mol] compared with 8.8% to 8.7% [73-72 mmol/mol] for RC (*P* = .03). Additionally, GEM^CGM^ reduced the need for diabetes medication (*P* = .01), reduced carbohydrate consumption (*P* = .009), and improved diabetes knowledge (*P* = .001), quality of life (*P* = .01) and diabetes distress (*P* = .02), and trended to more empowerment (*P* = .05) without increasing dietary fat, lipids, or hypoglycemia. Confirming our prior research, GEM^CGM^ appears to be a safe, effective lifestyle intervention option for adults with suboptimally controlled T2D who do not take insulin.

Pharmacological and lifestyle interventions are both important components of managing type 2 diabetes (T2D), but compared to the wide variety of pharmaceutical interventions with several different mechanisms of action, there are few lifestyle intervention options that are offered and they are often perceived to be difficult and complicated. The American Diabetes Association recommends 150 minutes per week of moderate physical activity plus 7% weight loss through caloric restriction [1]. Weight loss is not a viable option for patients who do not need or want to lose weight, are unable to lose weight, or cannot maintain weight loss. Consequently, lifestyle interventions with a different approach are needed. More recently, the American Diabetes Association stated: “Reducing overall carbohydrate intake for individuals with diabetes has demonstrated the most evidence for improving glycemia and may be applied in a variety of eating patterns” [2].

Previously, we reported on a new lifestyle intervention for T2D that, instead of focusing on reducing weight, shifts the focus to reducing postprandial glucose [3], which is a primary contributor to glycated hemoglobin (HbA_1c_) [4]. This program is called glycemic excursion minimization (GEM), which reflects its goal of minimizing postnutrient (meals, snacks, drinks) blood glucose (BG) excursions, or area under the curve (AUC). This initial study had 5 sessions of face-to-face GEM instruction and employed systematic BG monitoring [5]; it measured BG both before and 2 hours after meals and before and 30 minutes following exercise. BG feedback was used to *educate* participants regarding how different amounts and kinds of food and exercise affected their BG and to *motivate* them to repeat those choices that led to desirable BG levels. Participants were also encouraged to use BG feedback to *activate* them to make choices consistent with their current BG level and personal BG targets. For example, if participants discovered their BG was higher than desired before a meal, they could choose to postpone eating or take a brisk walk to lower their BG. However, the use of systematic BG was problematic because some people did not like pricking their finger, seeing blood, always having BG monitoring supplies available before and after eating and exercising, or trying to remember to monitor BG at specific times.

Continuous glucose monitoring (CGM) is an attractive alternative to systematic BG monitoring. CGM reduces the frequency of finger pricks, the need to remember equipment and when to take BG measurements, and it provides a more comprehensive BG record than systematic BG monitoring. These advantages could enhance the benefits of GEM [6]. Therefore, we hypothesized that relative to routine care (RC), GEM with CGM feedback (GEM^CGM^) would 1) significantly improve the primary outcome variables of HbA_1c_ and need for diabetes medication; 2) achieve these results through the mechanisms of increasing knowledge concerning food and activity choices, reducing consumption of carbohydrates, increasing routine physical activity, and diminishing BG excursions as reflected in CGM profiles; 3) have the secondary benefits of improving quality of life, empowerment, and diabetes distress; and 4) not have side effects like episodes of hypoglycemia [7] or increased compensatory fat consumption.

## 1. Materials and Methods

### A. Participants

Thirty adults with T2D were recruited through radio and print advertisements and the University of Virginia Hospital patient registry between July 2018 and August 2019. GEM^CGM^ training was conducted between December 2018 and September 2019, and 3-month follow-up assessments were performed between April 2019 and January 2020. Participants were between ages 30 and 80 years, had T2D for less than 11 years, had an HbA_1c_ greater than or equal to 7.0% (53 mmol/mol), were not on insulin or nondiabetic medications that could affect BG control (eg, prednisone), were able to walk for 30 minutes, and were interested in CGM. They were randomly assigned to RC or GEM^CGM^. Incentives to participate were free blood tests, blood glucose meters and supplies (Bayer Contour), activity monitors (Fitbit Charge 2), $100 on completion of postassessment, and CGM supplies if assigned to GEM^CGM^. The demographics of this sample appear in Table 1. The 2 groups did not significantly differ on any demographic variables.

**Table 1.** Demographic characteristics of routine care (RC) and glycemic excursion minimization plus real-time continuous glucose monitoring (GEM^CGM^) groups

	HbA_1c_, %	Duration of T2D, y	BMI, kg/m^2^	Female, %	White, %	Black, %	Age, y	Education, y
RC	8.8 ± 1.2	5.9 ± 2.5	35.6 ± 8.4	80	60	30	50.8 ± 14.2	16.1 ± 3.5
GEM^CGM^	8.9 ± 1.8	5.4 ± 2.7	33.5 ± 3.9	50	85	10	54.6 ± 12.2	14.8 ± 2.9

Abbreviations: BMI, body mass index; HbA_1c_, glycated hemoglobin; T2D, type 2 diabetes.

### B. Procedures

After being thoroughly informed, participants signed a University of Virginia institutional review board–approved consent form. Next, they participated in a baseline assessment that included a brief physical, blood tests for HbA_1c_ and lipids, a review of their current medications, and psychological questionnaires to assess attitudes toward glucose monitoring [8], quality of life [9], diabetes empowerment [10], diabetes distress [11], and depressive symptoms [12]. Using the Medication Effect Scale (MES) [13], the dose and type of each diabetes medicine in a participant’s medication regimen was converted to a common denominator—that medication’s average HbA_1c_-lowering potential. These potentials were then summed over the diabetes medications the individual was taking to estimate the potential HbA_1c_-lowering effect of a participant’s medication regimen. The following week participants wore a blinded activity monitor (Fitbit Charge 2), and were interviewed over the telephone on 2 work days and 1 weekend day to complete the automated self-administered 24-hour dietary recall dietary recall [14]. Ten RC and 12 GEM^CGM^ participants also wore a blinded CGM (Dexcom Platinum G4). This assessment was repeated a second time 5 months later—3 months after the conclusion of GEM^CGM^.

Following the baseline assessment, participants were randomly assigned, by a flip of a coin in blocks of 3 by the data manager, who was unfamiliar with the individual, to either GEM^CGM^ or RC in a 2:1 ratio; if “heads” came up during the first 2 participants, they were assigned to RC, and if “heads” did not come up then the third person was assigned to RC. All participants continued their usual care in consultation with their treating physician, who adjusted medication as clinically indicated throughout the 5-month study. One RC patient dropped out before postassessment to pursue bariatric surgery.

**Figure 1.**
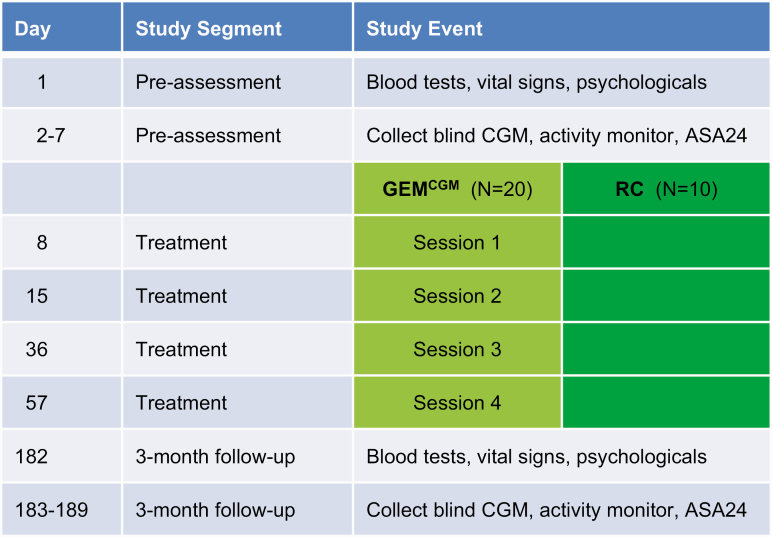
Timeline of study events. Glycemic excursion minimization plus real-time continuous glucose monitoring (GEM^CGM^) participants received one 7-day Dexcom G5 sensor at each of the 4 training sessions.

The 2-month GEM^CGM^ intervention period involved meeting in groups of 8 to 10 for 90 minutes on 4 occasions, with 1 week between sessions 1 and 2 and 3 weeks between sessions 2 and 3 and 3 and 4 (Fig. 1). At each session, participants were given a 7-day Dexcom G5 sensor, and 1 month after session 4, a fifth sensor was given. This timing was intended to diminish reliance on CGM and group support and to encourage autonomy following the conclusion of the intervention. Follow-up assessment occurred three months after session 4.

With the assistance of an instructor’s manual, a diabetes nurse educator (A.D.) led group sessions. GEM^CGM^ participants were given a 98-page manual and a diary that focused on the following:

- Session 1, Educating participants how their routine choices of types and amounts of food and physical activity affected their BG with the assistance of CGM feedback.
- Session 2, Reducing and eventually eliminating common high net carbohydrate foods with replacement and substitution, the results of which are reflected in CGM feedback. Participants were also instructed on the possibility and management of hypoglycemia.
- Session 3, Increasing mild and moderate physical activity, especially postprandial, both to increase direct glucose utilization and enhance insulin sensitivity. Again, CGM feedback was used to demonstrate the personal consequences/benefits of such choices.
- Session 4, Continuing and enhancing optimal food and activity choices over a lifetime and managing relapses.

At the beginning of each session, participants were asked to report the number of hypoglycemic experiences they had since the previous session, regarding level 1 (low enough for treatment), level 2 (sufficiently low to indicate serious, clinically important hypoglycemia), and level 3 (associated with severe cognitive impairment requiring external assistance for recovery) hypoglycemic events [7]. GEM^CGM^ participants were given 5 G5 sensors, 1 to insert at each session and 1 to be inserted 8 weeks before the 3-month follow-up assessment.

Three-month follow-up assessments were conducted between February 2019 and December 2019. Pre and post change scores for RC and GEM^CGM^ were compared in analyses of covariance, with baseline measures serving as the covariant. SPSS version 25 was used to perform the analyses.

## 2. Results

### A. Primary Outcome Variables

**Figure 2.**
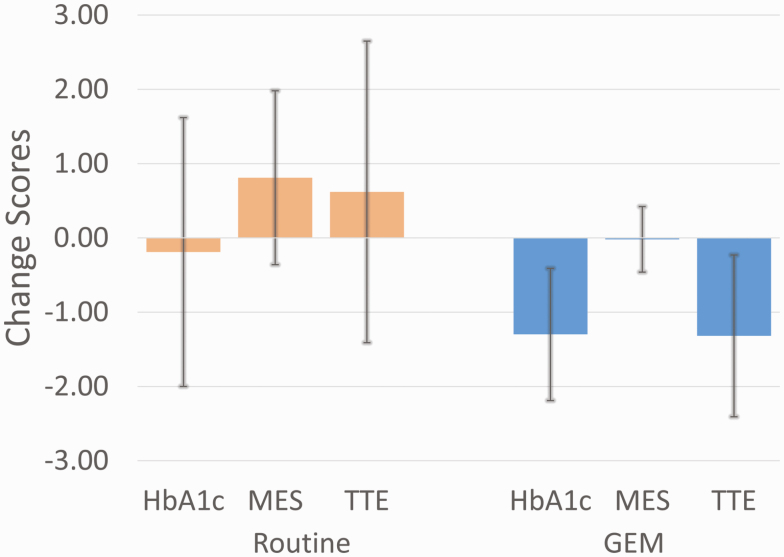
Means and SDs for glycated hemoglobin (HbA_1c_), medication effect score (MES), and total treatment effect (TTE) pre to post change scores.

Fig. 2 displays the mean and SD of pre and post change in HbA_1c_, MES, and the total treatment effect (TTE), which combines HbA_1c_ and MES as an HbA_1c_-equivalent impact of the intervention. GEM^CGM^ significantly reduced HbA_1c_ 1.11% more than RC and significantly reduced MES 0.83 more than RC. The combination of these, the TTE, separated RC from GEM^CGM^ by an HbA_1c_ equivalent of 1.94% (*P* < .001, see Fig. 2 and Table 2).

**Table 2.** Preassessment to postassessment change in study variables and statistical significance

Variable	Routine care, mean ± SD	GEM^CGM^, mean ± SD	*F*	*P*
Primary outcome variables				
HbA_1c_ (%)	–0.19 ± 1.81	–1.30 ± 0.89	5.57	.03
Medication effect score	0.81 ± 1.17	–0.02 ± 0.44	7.85	.009
Total treatment effect	0.62 ± 2.03	–1.32 ± 1.09	11.29	.000
Mechanisms				
Diabetes knowledge	–0.6 ± 2.4	3.7 ± 2.6	13.41	.001
Net carbohydrates (ASA24)	6.4 ± 112.5	–72.6 ± 75.8	8.17	.009
Average CDC active minutes (Fitbit)	–6.7 ± 36.0	4.5 ± 39.4	1.29	.27
Activity calories (Fitbit)	–22.7 ± 454.1	84.7 ± 454.3	0.60	.45
PHQ9 Depression symptoms	–1.4 ± 6.7	–2.3 ± 5.1	3.34	.08
Secondary variables				
WHO-QOL (Physiological)	0.1 ± 1.7	0.1 ± 1.4	1.35	.26
WHO-QOL (Psychological)	–0.8 ± 1.0	0.4 ± 1.6	6.99	.01
Glucose Monitor Satisfaction Survey	0.1 ± 0.6	0.5 ± 0.6	7.19	.01
Diabetes empowerment	0.9 ± 3.4	3.4 ± 4.8	4.36	.05
Diabetes Distress Scale (emotional)	0.2 ± 1.1	–0.5 ± 1.1	6.64	.02
Diabetes Distress Scale (regimen)	–0.1 ± 0.7	–0.9 ± 1.1	7.20	.01
Side effects				
Calories (ASA24)	–12 ± 957	–190 ± 719	3.68	.07
Total fat (ASA24)	3.2 ± 37.7	6.6 ± 37.4	2.92	.10
Saturated fat (ASA24)	–2.6 ± 13.4	2.6 ± 14.2	0.03	.87
Protein (ASA24)	–2.3 ± 56.2	10.4 ± 34.5	0.20	.66
LDL	–8.9 ± 31.9	1.1 ± 18.2	0.16	.70
HDL	–0.9 ± 7.0	2.8 ± 6.2	1.22	.28
Triglycerides	15.6 ± 98.4	–27.6 ± 62.6	1.48	.24
Total cholesterol	–5.3 ± 35.6	–3.3 ± 22.9	0.01	.93
Avg daily hypo AWC < 70 mg/dL, CGM	639 ± 1204	7 ± 22	1.07	.32

Abbreviations: ASA24, automated self-administered 24-hour dietary recall; AWC, area within the curve; CDC, Centers for Disease Control; CGM, continuous glucose monitoring; GEM^CGM^, glycemic excursion minimization plus real-time continuous glucose monitoring; HbA_1c_, glycated hemoglobin; HDL, high-density lipoprotein; LDL, low-density lipoprotein; PHQ9, Patient Health Questionnaire-9; WHO-QOL, World Health Organization Quality of Life.

Table 2 displays the pre and post means, SDs, *F* tests, and probabilities of the primary outcomes, mechanisms, secondary, and side effect variables.

### B. Mechanism Variables

As hypothesized, GEM^CGM^ did produce a significant increase in knowledge and led to a reduction in carbohydrate ingestion relative to RC. GEM^CGM^ did not significantly differ from RC in regards to increased physical activity or decreased glucose excursions (as quantified by AUC or time in range). However, change in the following variables did correlate with change in HbA_1c_: Fitbit calories burned (r = –0.40, *P* = .01) and minutes of daily moderate to vigorous activity (r = –0.37, *P* = .04); CGM AUC (r = 0.61, *P* = .009), and CGM time in range (r = –0.62, *P* = .006). Additionally, responsiveness to GEM^CGM^ was not limited by depressive symptoms because baseline Patient Health Questionnaire-9 (PHQ9) did not correlate with change in HbA_1c_ (r = –*0.13, P* = .50). This negative outcome was further confirmed when comparing participants with and without significantly elevated baseline PHQ9 scores (> 9) in regard to change in HbA_1c_, MES, or TTE (*P* = .51, .31, and .82, respectively).

### C. Secondary Outcome Variables

As hypothesized, GEM^CGM^ significantly improved psychological function relative to RC, including World Health Organization (WHO)-Quality of Life (Psychological subscale), Diabetes Empowerment, Diabetes Distress Scale (Emotional and Regimen subscales), and the Glucose Monitor Satisfaction Survey. It did not significantly improve WHO-Quality of Life (Physiological subscale).

### D. Side Effects

GEM^CGM^ did not lead to an increase in caloric, fat, or protein consumption nor a worsening in lipids relative to RC. Self-report experiences with hypoglycemia were not collected among RC participants. GEM^CGM^ participants reported 12 episodes of level 1 symptomatic hypoglycemia: 2 at session 2, 8 at session 3, and 2 at session 4. These were defined as “trembling, sweaty, pounding heart, unusual fatigue, pale skin, anxiety, irritability, with BG usually in the 60s, but it could be slightly higher or lower.” During the blinded CGM period, using the criterion that hypoglycemia events are defined as BG of 70 mg/dL or less [7], GEM and RC did not significantly differ regarding the pre and post change in the number of hypoglycemic events recorded.

## 3. Discussion

This study replicates our previous report [3] that found, relative to RC, that GEM led to a significant reduction in HbA_1c_ in adults with T2D. The present study extends those findings by demonstrating that when CGM was used instead of systematic BG monitoring, GEM^CGM^ reduced HbA_1c_ by a mean of 1.3% instead of the previous 1.0%, and did so with 20% fewer contacts (4 vs 5 training sessions) in a more diverse participant sample (longer diabetes duration, taking more varied diabetes medications). Further, the present study demonstrated that GEM^CGM^ reduced HbA_1c_ and MES more than RC (1.1% and 0.83, respectively), for a TTE difference of 1.94% HbA_1c_ equivalent between the 2 groups (see Fig. 2).

Compared to RC, GEM^CGM^ increased physical activity and decreased hyperglycemic excursions (AUC). However, these were not significantly different from RC. This was in part because the Dexcom G4 platinum captured only a maximum of 6.5 days of CGM data, the BG sampled in the HbA1c and blinded CGM measures did not overlap temporally (CGM followed the A_1c_), and the small sample size and large CGM SDs (see Table 3) restricted statistical power. However, changes in these parameters did significantly correlate with improvement in HbA_1c_, as did physical activity in a previous study [15]. Similarly to the previous study, GEM^CGM^ was associated with multiple psychological benefits compared to RC, including improvements in quality of life, greater sense of empowerment, and reduction in diabetes distress.

**Table 3.** Blinded continuous glucose monitoring (CGM) results. Percentage of time CGM was in different glucose ranges and CGM area under the curve (AUC) when blood glucose (BG) was greater than 180 mg/dL. No significant group differences were found

BG range, mg/dL	Routine care, pre	Routine care, post	GEM^CGM^, pre	GEM^CGM^, post
< 54	0%	1%	0%	0%
54-69	0%	1%	0%	0%
70-180	42%	43%	44%	50%
181-250	31%	29%	32%	27%
> 250	27%	26%	24%	23%
Average daily excursion AUC (> 180)	32 790 ± 32 775	35 942 ± 34 111	35 345 ± 46 259	33 336 ± 53 315

Abbreviation: GEM^CGM^, glycemic excursion minimization plus real-time continuous glucose monitoring.

These benefits were achieved without worsening lipids or increasing hypoglycemia. It is interesting to note that no GEM^CGM^ participants experienced level 2 hypoglycemia, whereas 2 RC participants, either having gone on insulin or glipizide, experienced 3 level 2 hypoglycemia events per patient during follow-up. This is reflected by an area within the curve under 70 mg/dL. RC showed a pre and post increase of 639 ± 1204, whereas GEM^CGM^ demonstrated an increase of 7 ± 22. These changes were not significantly different because of RC’s large variance.

Sample size is a significant limitation of the present study, potentially limiting the external validity of these findings and precluding investigation of individual differences. However, given replication of a previous study [3] with a different sample, any threat to external validity in the present study is diminished. The sample size was too small for the 2 groups to be matched on all variables. Although not significantly different, the RC group contained more female and fewer white participants. Furthermore, the sustainability of these findings is not certain given that the present study employed only a 3-month follow-up. Additionally, given that we recruited individuals interested in using CGM, these findings cannot be extrapolated to the broader T2D community, which includes people uninterested in CGM for a variety of reasons as noted previously. Finally, as in our previous study, which did not separate the effects of BG monitoring from the GEM didactic information, the present study does not separate any additive benefits of CGM from GEM didactics. This will be addressed in a future study.

These findings indicate that GEM^CGM^ is a viable option for adults with T2D interested in CGM, and gives diabetes clinicians an additional tool to aid consumers with T2D. It will be up to future studies to determine whether GEM^CGM^ is equivalent or superior to conventional weight loss therapy, which intervention is more sustainable, and which option best matches which patient characteristics.

## Data Availability

The data sets generated during and/or analyzed during the present study are not publicly available but are available from the corresponding author on reasonable request.

## Abbreviations

ASA24: automated self-administered 24-hour dietary recall
AUC: area under the curve
BG: blood glucose
CDC: centers for disease control
CGM: continuous glucose monitoring
GEM: glycemic excursion minimization
GEM^CGM^: glycemic excursion minimization plus real-time continuous glucose monitoring
HbA_1c_: glycated hemoglobin
MES: medication effect scale
PHQ9: Patient Health Questionnaire-9
RC: routine care
T2D: type 2 diabetes
TTE: total treatment effect
WHO: World Health Organization

## Additional Information

***Disclosure Summary:*** The authors have nothing to disclose.
