# CORRIGENDUM FOR “Minimizing Glucose Excursions (GEM) With Continuous Glucose Monitoring in Type 2 Diabetes: A Randomized Clinical Trial”

**DOI:** 10.1210/jendso/bvaa174

**Published:** 2020-11-06

In the above-named article by Cox DJ, Banton T, Moncrief M, Conaway M, Diamond A, and McCall A (Journal of the Endocrine Society 2020:4(11); doi: 10.1210/jendso/bvaa118), the following error occurred in the published paper: The wrong figure was provided for Figure 2. The correct Figure 2 has been replaced in the article online.

**Figure 2.**
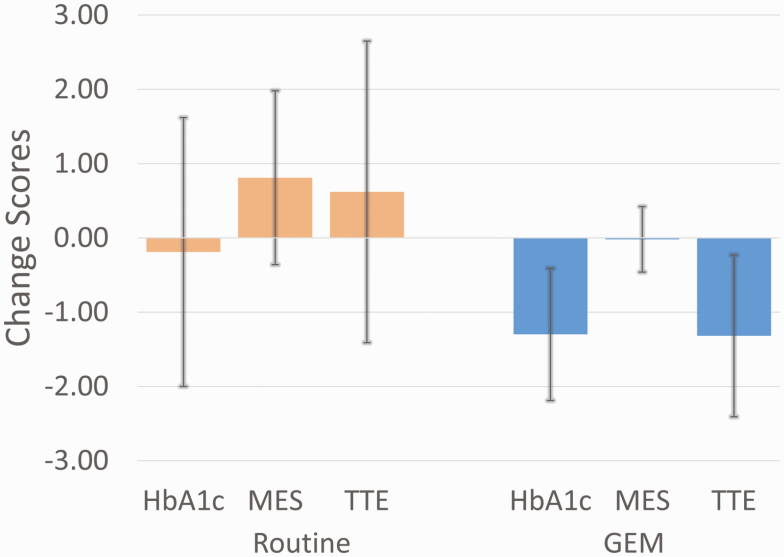


Doi: 10.1210/jendso/bvaa118

